# Priority-Aware Price-Based Power Control for Co-Located WBANs Using Stackelberg and Bayesian Games

**DOI:** 10.3390/s19122664

**Published:** 2019-06-13

**Authors:** Jingxian Wang, Yongmei Sun, Yuefeng Ji, Shuyun Luo

**Affiliations:** 1 State Key Laboratory of Information Photonics and Optical Communications, Beijing University of Posts and Telecommunications, Beijing 100876, China; wangjingxian@bupt.edu.cn (J.W.); jyf@bupt.edu.cn (Y.J.); 2 Department of Computer Science and Technology, Zhejiang Sci-Tech University, Hangzhou 310018, China; shuyunluo@zstu.edu.cn

**Keywords:** WBANs, interference, power control, Stackelberg game, Bayesian game

## Abstract

According to the IEEE 802.15.6 standard, interference within each wireless body area network (WBAN) can be well addressed by the time division multiple access (TDMA)-based media access control (MAC) protocol. However, the inter-WBAN interference will be caused after multiple WBANs are gathered together. This paper proposes a priority-aware price-based power control (PPPC) scheme for mitigating the inter-WBAN interference. Specifically, to maximize the transmission data rate of sensors and control the aggregate interference suffered by coordinators, a Stackelberg game is established, in which the coordinators issue interference prices and the active sensors adjust their transmission power accordingly. On the other hand, since the information about the identities of the active sensors in a specific time slot is kept private, a Bayesian game is designed to model the interaction among sensors. Moreover, the timeliness and reliability of data transmission are guaranteed by designing the sensors’ priority factors and setting a priority-related active probability for each sensor. At last, a power control algorithm is designed to obtain optimal strategies of game players. Simulation results show that compared with other existing schemes, the proposed scheme achieves better fairness with a comparable network sum data rate and is more energy efficient.

## 1. Introduction

As a promising solution for pervasive and remote health monitoring, wireless body area networks (WBANs) have attracted substantial attention in recent years. Generally, a WBAN consists of a set of biosensors that are implanted in or placed on or around the human body to collect the physiological parameters and a coordinator, e.g., a tablet or a smartphone, for gathering the sensed information and delivering it to remote medical centers via wireless communication technologies, such as WiFi and 4G [1,2,3,4]. Due to users’ mobile nature, WBANs are very likely to encounter one another. Thus, inter-WBAN interference will occur, which will degrade the intra-WBAN communication quality and quickly drain the sensors’ batteries [5]. This issue is more challenging in medical applications in which data transmission failure can be life-threatening.

Coexistence state prediction methods based on machine leaning models for co-located WBANs have been proposed in [6,7], which facilitate detection and processing of the inter-WBAN interference in time. Moreover, a few inter-WBAN interference mitigation schemes were proposed in previous works [8,9,10], especially those based on resource allocation, e.g., time slot assignment methods, channel allocation approaches, and power control schemes. Specifically, time slot assignment methods that adopt graph coloring algorithms were proposed in [11,12,13,14], in which the available time slots were mapped to colors and the adjacent nodes were assigned slots of different colors to avoid interference. The authors in [15,16,17,18] proposed channel allocation approaches, in which the interfering WBANs switched to different channels to alleviate the interference among WBANs. However, these methods will cause long time delays or co-channel interference when the number of co-located WBANs is large.

Power control schemes are considered effective methods of compensating for the aforementioned deficiencies [19]. The authors in [20,21] proposed power control schemes that were based on traditional non-cooperative game models. By adjusting the transmission power, the active sensor nodes attempt to maximize their own utilities, which are composed of their achievable data rate and consumed power. However, these methods are implemented based on the assumption that the private information about which sensor is active in the time slot of interest within a WBAN is exactly known by all its neighbors, which is not feasible in practice. On account of this, the authors in [22] proposed a Bayesian game mode-based power control (which we name BGPC in this paper) scheme for co-located WBANs, in which the WBANs act as players and the active links are taken as the types of players. The expected payoff of each player, which is defined as the difference between the throughput and the cost, where the cost is equal to the power price multiplied by the transmission power, is maximized. However, in the BGPC scheme, the power prices are fixed, and there is no dynamic pricing mechanism for controlling the aggregated interference at the coordinators.

Given all these considerations, we propose a priority-aware price-based power control (PPPC) scheme based on Stackelberg and Bayesian game models for inter-WBAN interference mitigation in this paper. Typically, the time division multiple access (TDMA) scheduling method is adopted within each WBAN to manage the intra-WBAN communication, while the information about which sensor is active in the time slot of interest is unknown to other WBANs. For the purpose of exposition, we assume there is a virtual player in each WBAN and that it takes the active sensor within the WBAN as its type. Briefly, the main intellectual contributions of this paper are summarized as follows:

- To control the power of the interference suffered by the coordinators and maximize the transmission data rate of sensors, a price-based power control scheme is proposed based on the Stackelberg game, in which the coordinators act as leaders by setting optimal interference prices, whereas the virtual players act as followers by adjusting their transmission power according to the received prices. In addition, both the leader-level game and the follower-level game are based on the non-cooperative game structure, in which the players aim to maximize their individual utilities selfishly.
- Due to the incomplete information caused by the privacy of time allocation in each WBAN, the competition among followers is modeled by a Bayesian game, in which each virtual player adopts different strategies for different types to maximize its own expected payoff.
- The sensors’ priorities are guaranteed by introducing the priority factors into the utility functions. Furthermore, the active probability of each sensor is proportional to its priority factor, to improve the timeliness and reliability of critical data transmission and prolong the network lifetime.
- A power control algorithm is designed to obtain the optimal strategies of game players. Simulations are conducted to evaluate the effectiveness of the proposed PPPC scheme in terms of the total network data rate, energy efficiency, and fairness among sensors by comparing with other existing schemes.

The remainder of this paper is organized as follows: Section 2 presents a brief review of the related work. Section 3 provides the system model and problem formulation. The proposed priority-aware price-based power control scheme is analyzed in Section 4. Section 5 evaluates the performance of the proposed scheme. Finally, the conclusions of the paper are presented in Section 6. The important notations used in this paper are provided in Table 1.

## 2. Related Work

Inter-WBAN interference results in decreased network performance and quick energy consumption. Power control schemes that have the capability of mitigating the interference and improving the energy efficiency have been studied in many works. The authors in [20,21,23,24,25] proposed power control schemes based on the traditional non-cooperative game model, in which the active nodes determine their transmission powers selfishly to maximize their own utilities, which are composed of the transmission rate and the required power. Specifically, a reinforcement learning method was introduced in [23] to allow WBANs to improve their performance by learning from experience. A quality of service (QoS)-driven power control approach was proposed in [24], in which the satisfaction degree of each sensor with its signal-to-interference plus noise ratio (SINR) and the energy consumption are considered in the utility function. Additionally, the power control scheme proposed in [25] was based on the users’ interaction information, in which Bluetooth and acoustic wave technologies are used to estimate the distance between WBANs. Moreover, in our previous work [26], a QoS-aware power control scheme based on the Nash bargaining game model was proposed, where the interfering nodes adjust their transmission powers cooperatively according to the diverse QoS requirements.

In the aforementioned power control schemes, there is no dynamic pricing mechanism for controlling the power of the interference suffered by the coordinators. As an effective tool for formulating the pricing mechanism, the Stackelberg game model [27] has been implemented in many other fields. The authors in [28,29] studied the implementation of the Stackelberg game model in cooperative communication networks, in which the relay nodes set prices and get paid for helping users forward signals and the sources pay for the power of the relay nodes. Stackelberg game-based power allocation schemes for femtocell networks were proposed in [30,31,32], where the macrocell base station protects itself by pricing the interference from femtocell users. In cellular networks, the operators set an interference penalty price for each user to avoid intolerable interference at the WiFi access point, which can be formulated by the Stackelberg game model [33]. The authors in [34] formulated a Stackelberg game model for capturing the interactions between the energy management centers and the devices in smart grids, where the former offer virtual retail prices and the latter are supposed to purchase energy. Additionally, the authors in [35,36] studied the Stackelberg game model-based incentive mechanism in peer-to-peer networks.

However, these methods assume that each player has exact information about the other players in the network, which may not be feasible in practice. To solve this problem, a few previous works [22,37,38,39] have studied the application of the Bayesian game in wireless networks with uncertainty. Specifically, Stackelberg and Bayesian game model-based power control schemes have been proposed for an anti-jamming network [38] and a two-tier cellular network [39]. However, these schemes cannot be applied to WBANs directly due to the specific features of WBANs. Thus, we propose a tailored Stackelberg and Bayesian game-based power control scheme for interference mitigation in co-located WBANs, in which the distinct parameters of sensors are considered to improve the network QoS.

## 3. System Model and Problem Formulation

In this section, we first introduce the system model, including the interference model and the channel model. Then, the problem formulation based on the Stackelberg and Bayesian game models is presented.

### 3.1. System Model

**Figure 1:**
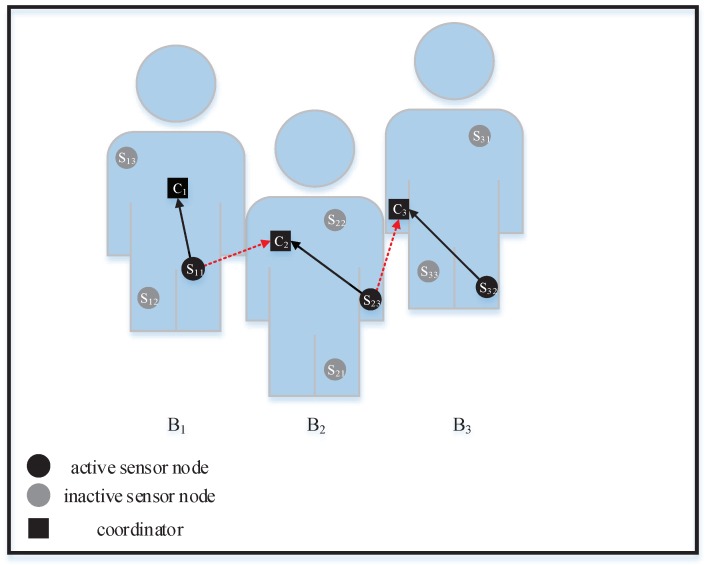
Interference model among co-located WBANs.

In this paper, we consider a spectrum-sharing scenario with *N* co-located WBANs, which is denoted by B = {$B_{1}$, $B_{2}$, …, $B_{N}$}, where $B_{i}$ represents the $i^{\mathrm{th}}$ WBAN. Within each WBAN, there is a star topology, which consists of *m* sensor nodes that measure the physiological parameters of the human body, such as EEG, ECG, and body temperature, and a coordinator for collecting the sensed data from its sensors. The sets of sensors and coordinators are denoted by S = $\left\{\left.S_{ij}\right|i=1,2,\ldots ,N,j=1,2,\ldots ,m\right\}$ and C = $\left\{\left.C_{i}\right|i=1,2,\ldots ,N\right\}$, respectively, where $S_{ij}$ denotes the $j^{\mathrm{th}}$ sensor in the $i^{\mathrm{th}}$ WBAN and $C_{i}$ is the coordinator of the $i^{\mathrm{th}}$ WBAN. We assume that the TDMA scheduling is introduced within each WBAN to mitigate the intra-WBAN interference. However, inter-WBAN interference may be incurred by sensors that are working simultaneously in co-located WBANs. The interference model is illustrated in Figure 1. We focus on the uplink communication in this paper. Specifically, there are two types of links, namely the on-body intended link between a sensor and its corresponding coordinator and off-body interference links between different WBANs, which are denoted by the solid lines and dotted lines, respectively.

Without loss of generality, we assumed that the involved channels were block-fading; i.e., the channels are invariant in each time slot, but may vary across successive slots. The channel gain of the link from sensor $S_{ij}$ to coordinator $C_{k}$ is denoted by $h_{ij}^{k}$, which is a function of the distance between the transceivers. Thus, the SINR of sensor $S_{ij}$ can be formulated as follows:

(1) $$\gamma_{ij}=\frac{p_{ij}h_{ij}^{i}}{I_{i}+N_{0}}$$

where $p_{ij}$ is the transmission power of sensor $S_{ij}$, $I_{i}$ represents the power of aggregated interference that is suffered by coordinator $C_{i}$, and $N_{0}$ denotes the Gaussian white noise power. Then, based on Shannon’s formula, the maximum transmission data rate of sensor $S_{ij}$ is given by:

(2) $$\varphi_{ij}=W\log_{2}\left(1+\gamma_{ij}\right)$$

where *W* indicates the available bandwidth.

### 3.2. Problem Formulation

Since the co-located WBANs work non-cooperatively, the time allocation within each WBAN is private information that is unknown to others; i.e., the WBANs have no knowledge about which nodes are selected in other WBANs to transmit in the time slot of interest. Given the incomplete information, a Bayesian game is employed, in which players adopt different strategies for different types. In this paper, we assume there is a virtual player in each WBAN with *m* types. Thus, the game can be characterized as follows:

- The set of the virtual players is denoted as $V=\left\{\left.V_{i}\right|i=1,2,\ldots ,N\right\}$
- The type set of player $V_{i}$ consists of the *m* sensors within the $i^{\mathrm{th}}$ WBAN and is denoted by $T_{i}$, where $t_{ij}=S_{ij}$, $t_{ij}\in T_{i}$ implies that sensor $S_{ij}$ is active in the considered time slot.
- The strategy of player $V_{i}$ is its transmission power, which is a function of its type. Specifically, $p_{ij}$ is player $V_{i}$’s transmission power when its type is $t_{ij}=S_{ij}$. The strategy set of player $V_{i}$ is $P_{i}$, i.e., $p_{ij}\in P_{i},\forall j$.
- The probability that $S_{ij}$ is active in the considered time slot is $Pr_{ij}$, which is common knowledge among all players.

Each sensor tries to maximize its own data rate selfishly by increasing its transmission power, which will result in quick energy depletion of the sensor and cause severe interference to other WBANs that are active simultaneously. To guarantee the network QoS, the interference pricing mechanism is employed, in which the coordinators have the privilege of taking actions first to set the interference prices to maximize their profits. Then, the active sensors update their transmission powers according to the received prices to maximize their payoffs. The two-stage game can be formulated by the Stackelberg game model, where the coordinators act as the leaders and the virtual players are the followers.

Specifically, as the types of followers within a particular time slot are unknown, the profit of leader $C_{i}$ is given by:

(3) $${U_{i}}^{L}=\sum_{N_{-i}}\rho_{i}I_{i}Pr\left(N_{-i}\right)$$

where $\rho_{i}$ is the interference price that is set by $C_{i}$, $N_{-i}$ is a stochastic set that is composed of the active sensors in all WBANs except $B_{i}$, and $Pr\left(N_{-i}\right)$ is the occurrence probability of concurrently transmitting set $N_{-i}$.

Moreover, the expected payoff of virtual player $V_{i}$ is given by:

(4) $$\begin{array}{c} {U_{i}}^{F}=\sum_{j=1}^{m}u_{ij}Pr_{ij}\\ =\sum_{j=1}^{m}\left\{\sum_{N_{-i}}\left[\ln\left(1+\vartheta_{ij}p_{ij}h_{ij}^{i}\right)-I_{i}-\sum_{k=1,k\neq i}^{N}p_{ij}h_{ij}^{k}\rho_{k}\right]Pr\left(N_{-i}\right)\right\}Pr_{ij} \end{array}$$

where $u_{ij}$ is the payoff of sensor $S_{ij}$ and $\vartheta_{ij}$ is the priority factor of sensor $S_{ij}$. Referring to [26], $\vartheta_{ij}$ can be defined as follows:

(5) $$\vartheta_{ij}=e^{\frac{\left|\zeta_{ij}-\zeta_{ij,0}\right|}{\zeta_{ij,0}}}-\frac{E_{ij}}{E_{0}}$$

where $\zeta_{ij}$ is the sensed value of a particular physiological signal, $\zeta_{ij,0}$ is the corresponding normal value of the signal, $E_{ij}$ is the energy that has been consumed by sensor $S_{ij}$, and $E_{0}$ is the initial energy. The first term of $\vartheta_{ij}$ ($e^{\frac{\left|\zeta_{ij}-\zeta_{ij,0}\right|}{\zeta_{ij,0}}}$) indicates the abnormality of the data sensed by $S_{ij}$. The second term of $\vartheta_{ij}$ ($\frac{E_{ij}}{E_{0}}$) reflects the energy efficiency of $S_{ij}$.

In Formula (4), the first term ($\ln(1+\vartheta_{ij}p_{ij}h_{ij}^{i})$) estimates the benefit obtained by sensor $S_{ij}$, which provides an incentive for the sensor to enhance its transmission power level. The second term ($I_{i}$) captures the negative impact that other sensors’ strategies have on $S_{ij}$. Finally, the last term ($\sum_{k=1,k\neq i}^{N}p_{ij}h_{ij}^{k}\rho_{k}$) represents the cost that $S_{ij}$ has to pay for generating interference with other WBANs.

## 4. Analysis of the Priority-Aware Price-Based Power Control Scheme

The backward induction method was employed to analyze the PPPC scheme. That is, the followers first maximize their utilities by adjusting their transmission powers based on any given interference prices. Then, the leaders set optimal interference prices according to the perceived responses of the followers. Thus, this section analyzes both the follower-level game and the leader-level game and presents the implementation of the PPPC scheme.

### 4.1. Follower-Level Game

Based on the prices issued by the leaders, the followers compete with one another non-cooperatively to maximize their individual payoffs. Because of the incomplete information, the competition among followers is modeled by a Bayesian game. The Bayesian Nash equilibrium (BNE) is the solution of the Bayesian game, which is defined as a mapping from the type set to the strategy set, i.e., $f_{i}:T_{i}\to P_{i},\forall i$. To achieve the BNE of the follower game, the following optimization problem should be solved:

P1:

(6) $$\begin{array}{c} \max\;{U_{i}}^{F}\\ s.t.\;0\leq p_{ij}\leq P_{\max},\forall j \end{array}$$

where $P_{\max}$ is the maximum transmission power of sensors. Because the active probability of each sensor is non-negative, i.e., $Pr_{ij}\geq 0,\forall i,j$, and the sensors within a WBAN determine their strategies independently, the above problem can be simplified as follows:

P2:

(7) $$\begin{array}{c} \max\;u_{ij}\\ s.t.\;0\leq p_{ij}\leq P_{\max} \end{array}$$

Moreover, since the co-located WBANs work independently, the payoff of $S_{ij}$ can be rewritten as:

(8) $$u_{ij}=\ln\left(1+\vartheta_{ij}p_{ij}h_{ij}^{i}\right)-\sum_{k=1,k\neq i}^{N}\sum_{j=1}^{m}p_{kj}h_{kj}^{i}Pr_{kj}-\sum_{k=1,k\neq i}^{N}p_{ij}h_{ij}^{k}\rho_{k}$$

**Proposition** **1.** *The best response of follower $V_{i}$ when performing action $p_{ij}$ is:*

(9) $$p_{ij}=\frac{1}{\sum_{k=1,k\neq i}^{N}h_{ij}^{k}\rho_{k}+\lambda_{ij}-\tau_{ij}}-\frac{1}{\vartheta_{ij}h_{ij}^{i}}$$

**Proof.**  The second-order derivative of $u_{ij}$ with respect to $p_{ij}$ is given as:

(10) $$\frac{\partial^{2}u_{ij}}{\partial {p_{ij}}^{2}}=-\frac{{\left(\vartheta_{ij}h_{ij}^{i}\right)}^{2}}{{\left(1+\vartheta_{ij}p_{ij}h_{ij}^{i}\right)}^{2}}\leq 0$$

Thus, problem P2 is a convex optimization problem. The unique optimal solution can be achieved using the Lagrange multiplier method. The Lagrange function is given by:

(11) $$L=\ln\left(1+\vartheta_{ij}p_{ij}h_{ij}^{i}\right)-\sum_{k=1,k\neq i}^{N}\sum_{j=1}^{m}p_{kj}h_{kj}^{i}Pr_{kj}-\sum_{k=1,k\neq i}^{N}p_{ij}h_{ij}^{k}\rho_{k}-\lambda_{ij}\left(p_{ij}-P_{\max}\right)+\tau_{ij}p_{ij}$$

Taking the first-order derivative of (11) with respect to $p_{ij}$ and setting it to zero, we obtain the following equation:

(12) $$\frac{\partial L}{\partial p_{ij}}=\frac{\vartheta_{ij}h_{ij}^{i}}{1+\vartheta_{ij}p_{ij}h_{ij}^{i}}-\sum_{k=1,k\neq i}^{N}h_{ij}^{k}\rho_{k}-\lambda_{ij}+\tau_{ij}=0$$

Thus, the optimal solutions of followers can be derived. □

It can be observed from Formula (9) that a sensor with a larger priority factor will enhance its transmission power to improve the reliability and timeliness of data transmission. In contrast, a sensor with a smaller priority factor will decrease its transmission power to save energy. Moreover, a sensor will lower its transmission power when the received interference prices are higher to decrease its cost.

### 4.2. Leader-Level Game

The leaders get paid for suffering interference that is generated by followers in other WBANs. Based on Formulas (3) and (9), the profit of coordinator $C_{i}$ can be reformulated as:

(13) $$\begin{array}{c} {U_{i}}^{L}=\rho_{i}\sum_{k=1,k\neq i}^{N}\sum_{j=1}^{m}p_{kj}h_{kj}^{i}Pr_{kj}\\ =\rho_{i}\sum_{k=1,k\neq i}^{N}\sum_{j=1}^{m}\left(\frac{1}{\sum_{q=1,q\neq k}^{N}h_{kj}^{q}\rho_{q}+\lambda_{kj}-\tau_{kj}}-\frac{1}{\vartheta_{kj}h_{kj}^{k}}\right)h_{kj}^{i}Pr_{kj} \end{array}$$

In the leader-level game, each leader aims at maximizing its own profit, which can be expressed as follows:

P3:

(14) $$\begin{array}{c} \max\;{U_{i}}^{L}\\ s.t.\;\rho_{i}\geq 0 \end{array}$$

**Proposition** **2.** *The best response of leader $C_{i}$ is given by:*

(15) $$\rho_{i}=\frac{\sum_{k=1,k\neq i}^{N}\sum_{j=1}^{m}\left(\frac{1}{\sum_{q=1,q\neq k}^{N}h_{kj}^{q}\rho_{q}+\lambda_{kj}-\tau_{kj}}-\frac{1}{\vartheta_{kj}h_{kj}^{k}}\right)h_{kj}^{i}Pr_{kj}+\eta_{i}}{\sum_{k=1,k\neq i}^{N}\sum_{j=1}^{m}\left[\frac{{\left(h_{kj}^{i}\right)}^{2}Pr_{kj}}{{\left(\sum_{q=1,q\neq k}^{N}h_{kj}^{q}\rho_{q}+\lambda_{kj}-\tau_{kj}\right)}^{2}}\right]}$$

**Proof.**  It can be proven that the objective function of P3 is a concave function of $\rho_{i}$, i.e., $\frac{\partial^{2}{U_{i}}^{L}}{\partial {\rho_{i}}^{2}}<0$. Thus, problem P3 is a convex optimization problem and can be solved by the Lagrange multiplier method, where the Lagrange function is expressed as follows:

(16) $$L_{0}=\rho_{i}\sum_{k=1,k\neq i}^{N}\sum_{j=1}^{m}\left(\frac{1}{\sum_{q=1,q\neq k}^{N}h_{kj}^{q}\rho_{q}+\lambda_{kj}-\tau_{kj}}-\frac{1}{\vartheta_{kj}h_{kj}^{k}}\right)h_{kj}^{i}Pr_{kj}+\eta_{i}\rho_{i}$$

Taking the first-order derivative of Formula (16) with respect to $\rho_{i}$ and setting it to zero, the following equation can be obtained:

(17) $$\frac{\partial L_{0}}{\partial \rho_{i}}=\sum_{k=1,k\neq i}^{N}\sum_{j=1}^{m}\left(\frac{\sum_{q=1,q\neq k}^{N}h_{kj}^{q}\rho_{q}+\lambda_{kj}-\tau_{kj}-\rho_{i}h_{kj}^{i}}{{\left(\sum_{q=1,q\neq k}^{N}h_{kj}^{q}\rho_{q}+\lambda_{kj}-\tau_{kj}\right)}^{2}}-\frac{1}{\vartheta_{kj}h_{kj}^{k}}\right)h_{kj}^{i}Pr_{kj}+\eta_{i}=0$$

Then, the optimal solutions of coordinators can be derived. □

### 4.3. Implementation of the Proposed PPPC Scheme

To avoid encountering the NP-hard problem that results from using the traditional optimization algorithms, the fixed-point method is applied to solve the proposed problem [27]. The iteration steps are as follows:

(18) $$\rho_{i}\left(t+1\right)=\frac{\sum_{k=1,k\neq i}^{N}\sum_{j=1}^{m}\left(\frac{1}{\sum_{q=1,q\neq k}^{N}h_{kj}^{q}\rho_{q}\left(t\right)+\lambda_{kj}\left(t\right)-\tau_{kj}\left(t\right)}-\frac{1}{\vartheta_{kj}h_{kj}^{k}}\right)h_{kj}^{i}Pr_{kj}+\eta_{i}\left(t\right)}{\sum_{k=1,k\neq i}^{N}\sum_{j=1}^{m}\left[\frac{{\left(h_{kj}^{i}\right)}^{2}Pr_{kj}}{{\left(\sum_{q=1,q\neq k}^{N}h_{kj}^{q}\rho_{q}\left(t\right)+\lambda_{kj}\left(t\right)-\tau_{kj}\left(t\right)\right)}^{2}}\right]},\forall i$$

(19) $$\lambda_{ij}\left(t+1\right)=\max\left\{\lambda_{ij}\left(t\right)+\left[\frac{1}{\sum_{k=1,k\neq i}^{N}h_{ij}^{k}\rho_{k}\left(t\right)+\lambda_{ij}\left(t\right)-\tau_{ij}\left(t\right)}-\frac{1}{\vartheta_{ij}h_{ij}^{i}}-P_{\max}\right],0\right\},\forall i,j$$

(20) $$\tau_{ij}\left(t+1\right)=\max\left\{\tau_{ij}\left(t\right)-\left[\frac{1}{\sum_{k=1,k\neq i}^{N}h_{ij}^{k}\rho_{k}\left(t\right)+\lambda_{ij}\left(t\right)-\tau_{ij}\left(t\right)}-\frac{1}{\vartheta_{ij}h_{ij}^{i}}\right],0\right\},\forall i,j$$

(21) $$\eta_{i}\left(t+1\right)=\max\left\{\eta_{i}\left(t\right)-\rho_{i}\left(t\right),0\right\},\forall i$$

where *t* denotes the iteration number.

Specifically, to improve the timeliness of critical data transmission, we assume that the active probability of each sensor is proportional to its priority factor, which is defined as follows:

(22) $$Pr_{ij}=\frac{\vartheta_{ij}}{\sum_{l=1}^{m}\vartheta_{il}},\forall i,j$$

Here, a power control algorithm is designed to implement the proposed PPPC scheme, as described in Algorithm 1.

**Algorithm 1** Power control algorithm.
1:**Input**: $h_{kj}^{q}$, $\vartheta_{kj}$, $Pr_{kj}$, $\lambda_{kj}\left(t\right)$, $\tau_{kj}\left(t\right)$, $\eta_{i}\left(t\right)$, $\rho_{q}\left(t\right)$, k=1⋯N,k≠i, q=1⋯N,q≠k, j=1⋯m, i=1⋯N2:**Output**: optimal interference prices, transmission power3:**Initialization**: $\rho_{q}\left(t\right)=0.5$, $\lambda_{kj}\left(t\right)=\tau_{kj}\left(t\right)=\eta_{i}\left(t\right)=10$4:Compute the $\rho_{i}\left(t+1\right)$, $\lambda_{ij}\left(t+1\right)$, $\tau_{ij}\left(t+1\right)$ and $\eta_{i}\left(t+1\right)$ according to Formulas (18)–(21)5:**While**∃i, $|\rho_{i}\left(t+1\right)-\rho_{i}\left(t\right)|>\varepsilon$, **do**6:
$\rho_{i}\left(t\right)=\rho_{i}\left(t+1\right)$
7:
$\lambda_{ij}\left(t\right)=\lambda_{ij}\left(t+1\right)$
8:
$\tau_{ij}\left(t\right)=\tau_{ij}\left(t+1\right)$
9:
$\eta_{i}\left(t\right)=\eta_{i}\left(t+1\right)$
10:Update $\rho_{i}\left(t+1\right)$, $\lambda_{ij}\left(t+1\right)$, $\tau_{ij}\left(t+1\right)$, $\eta_{i}\left(t+1\right)$ according to Formulas (18)–(21)11:
**end**
12:$\rho_{i}\left(t+1\right),\forall i$ is the optimal interference prices, and compute the transmission power based on Formula (9)


## 5. Performance Evaluation

In this section, the simulation results are presented. The simulation was designed on the MATLAB platform. We set up a network with *N* WBANs that were randomly deployed in a 1.6 m × 1.4 m rectangular area (Plane size of the passenger elevator car). For simplicity, each WBAN was mapped to a rectangle with length 0.5 m and width 0.3 m [26]. In each WBAN, there were two sensors, i.e., m=2, which were randomly placed in the rectangle, and a coordinator was placed in the center of the rectangle for effective communication with its sensors.

In the simulation, as an example, the channel gain $h_{ij}^{k}={\left(d_{ij}^{k}\right)}^{-2}$, where $d_{ij}^{k}$ is the distance between sensor $S_{ij}$ and coordinator $C_{k}$ [22], the maximum transmission power $P_{\max}=0\;\mathrm{dBw}$, and the available bandwidth W=4 kHz. The parameters in the simulation are listed in Table 2, and the priority factor of each sensor, which is generated randomly, is given in Table 3.

For comparison, the following schemes were simulated:

OPTIMALscheme [29]: it aims to maximize the network sum data rate.

EVENscheme [39]: the sensors within a WBAN are activated with equal probability.

BGPC scheme [22]: the Bayesian game-based power control scheme with a fixed interference price.

### 5.1. Feasibility of the PPPC Scheme

**Figure 2:**
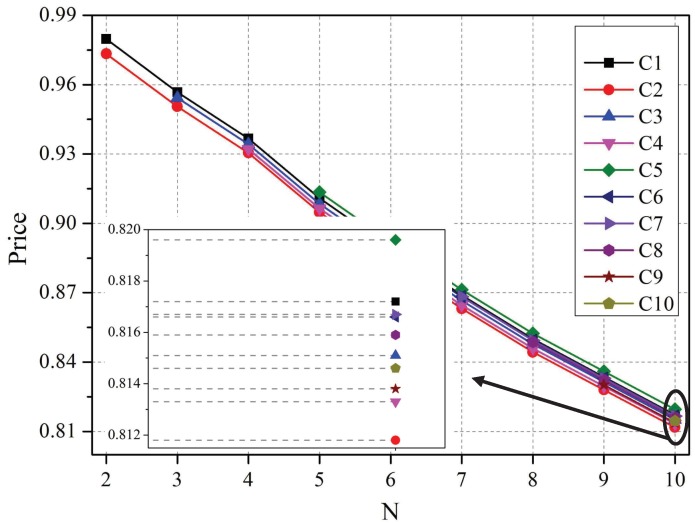
Optimal strategies of leaders vs. the number of co-located WBANs.

**Figure 3:**
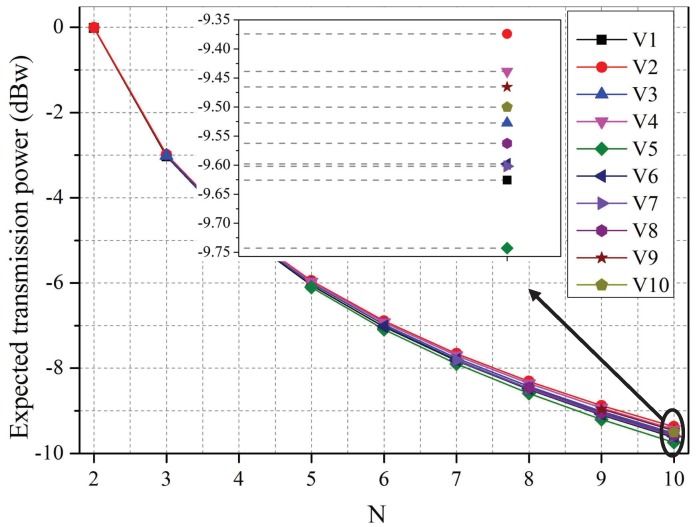
Optimal strategies of followers vs. the number of co-located WBANs.

Figure 2 and Figure 3 show the players’ optimal strategies as the number of co-located WBANs increases from 2–10. As more WBANs become clustered together, the competition among them becomes increasingly fierce. In this case, according to Figure 2, each leader decreased its price to maximize its profit, which complies with the rules in an economic market, and each follower lowered its expected transmission power to decrease its total cost, as depicted in Figure 3.

Specifically, based on the knowledge of the best responses of sensors, the coordinators understand that the sensor with a larger priority factor is certain to increase its transmission power to enhance the received signal strength. Therefore, the neighbor coordinators of the sensor will raise their interference prices to obtain more profits, as illustrated in Figure 2.

It can be observed from Figure 3, though the received prices are higher, the sensor with a higher priority level will increase its transmission power at any cost to improve the reliability and timeliness of abnormal data transmission, which is applicable to WBAN collecting life-critical physiological data.

Mathematically, the above phenomena can be analyzed based on Formulas (9) and (15).

**Figure 4:**
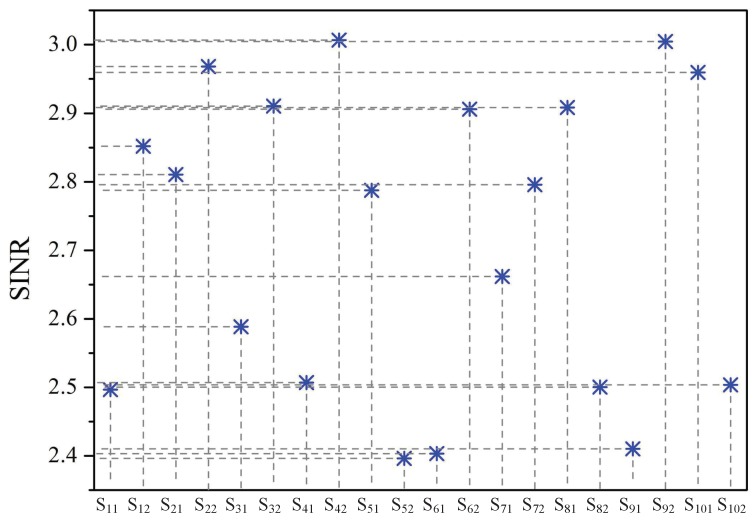
SINR of each sensor.

SINR reflects the timeliness and reliability of data transmission [40]. Figure 4 depicts the SINRs of sensors with different priority levels when there were 10 co-located WBANs. It can be seen that there is a positive correlation between the sensors’ priority factors and their obtained SINRs. When the sensed data were abnormal, that is when the corresponding sensor had a large priority factor, it would enhance its SINR by increasing its transmission power to improve the timeliness and reliability of critical data transmission. In contrast, when the sensor had consumed much energy, that is when it had a small priority factor, the sensor would lower its transmission power to prolong its lifetime, which resulted in decreased SINR.

**Figure 5:**
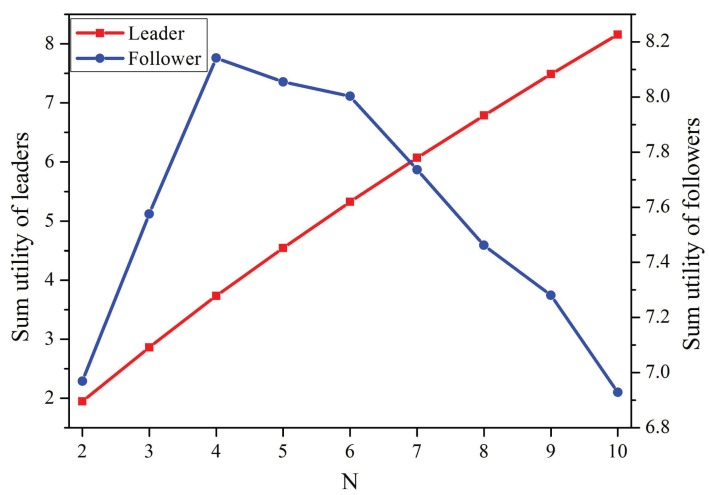
Sum utility of players vs. the number of co-located WBANs.

Figure 5 shows the sum utilities of the leaders and followers as functions of the number of co-located WBANs. The sum utility of the leaders increased as more WBANs joined the network. Moreover, when the number of co-located WBANs increased from 2–4, the sum utility of the followers increased. However, the sum utility of the followers decreased when there were more than four WBANs. The reason is that the followers must pay more leaders and suffer from more severe interference in this case, which decreased their utilities dramatically.

**Figure 6:**
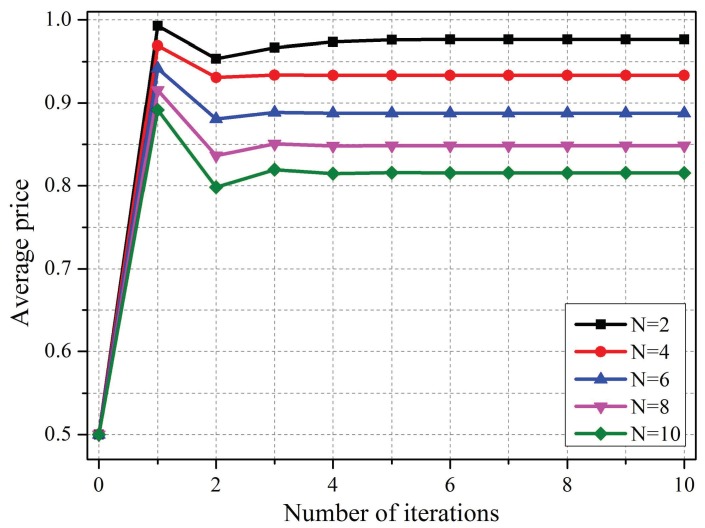
Convergence performance of the proposed scheme.

In the proposed PPPC scheme, the optimal strategies of coordinators were achieved iteratively, as analyzed in Section 4.3. According to Figure 6, the interference prices that were set by coordinators would converge quickly under scenarios with different numbers of co-located WBANs, which indicates the feasibility of the proposed scheme.

### 5.2. Comparison of PPPC with Other Schemes

**Figure 7:**
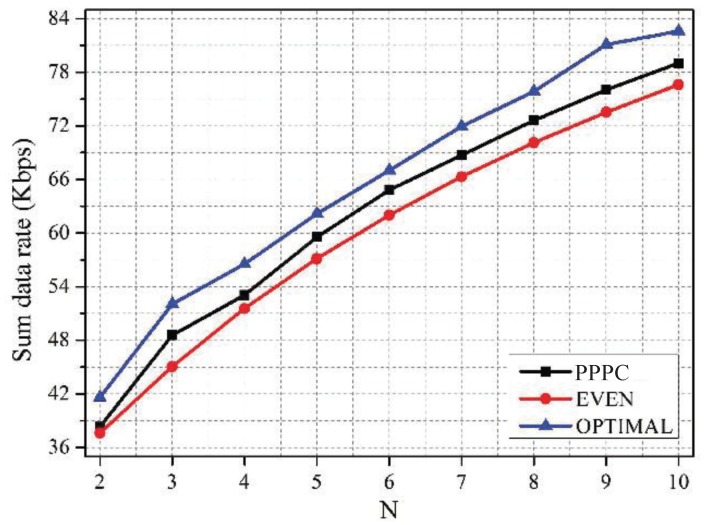
Sum data rate vs. *N* under schemes with different active probabilities of sensors. PPPC, priority-aware price-based power control.

**Figure 8:**
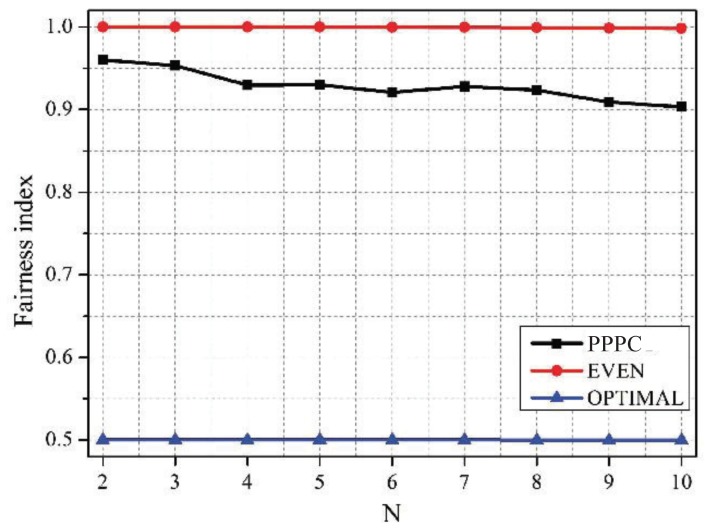
Fairness among sensors vs. *N* under schemes with different active probabilities of sensors.

Figure 7 and Figure 8 compare the proposed PPPC scheme with the EVEN scheme and the OPTIMAL scheme in terms of the network sum data rate and the fairness among sensors. Specifically, the fairness among sensors was quantified by Jain’s fairness index [41], which is defined as:

(23) $$\mathrm{Fairness}\;\mathrm{index}=\frac{{\left(\sum_{i=1}^{N}\sum_{j=1}^{m}x_{ij}\right)}^{2}}{Nm\sum_{i=1}^{N}\sum_{j=1}^{m}x_{ij}^{2}}$$

where $x_{ij}$ is the achievable data rate of sensor $S_{ij}$.

It can be figured out from Figure 7 that the PPPC scheme outperformed the EVEN scheme in terms of the data rate by 3.5%, on average. Although the sum data rate of the OPTIMAL scheme was 5.05% higher than that of the PPPC scheme, the OPTIMAL scheme resulted in the smallest fairness index, as shown in Figure 8. The reason is that the OPTIMAL scheme neglected the requirement of sensors for fairness, while maximizing the sum data rate. Conversely, the EVEN scheme aimed to guarantee the fairness by sacrificing the data rate. Thus, the fairness of the EVEN scheme was slightly higher than that of the PPPC scheme. However, the proposed PPPC scheme achieved a good tradeoff between the network sum utility and the fairness among sensors by setting the priority-level-related active probability for each sensor.

**Figure 9:**
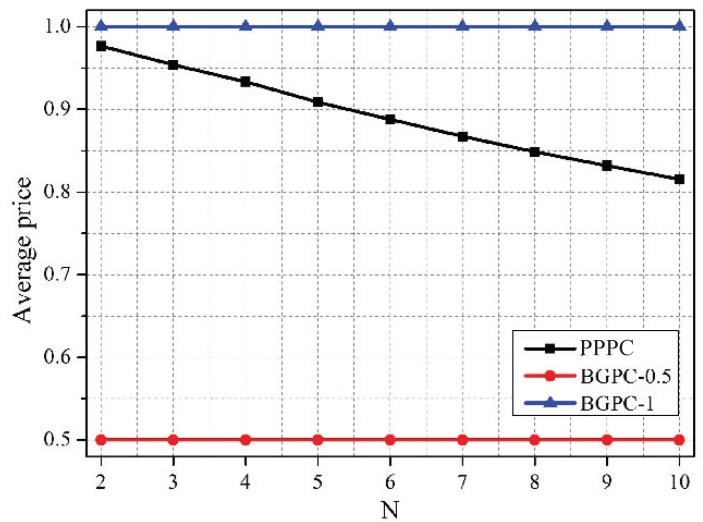
Average prices vs. *N* under schemes with different pricing mechanisms. BGPC, Bayesian game mode-based power control.

**Figure 10:**
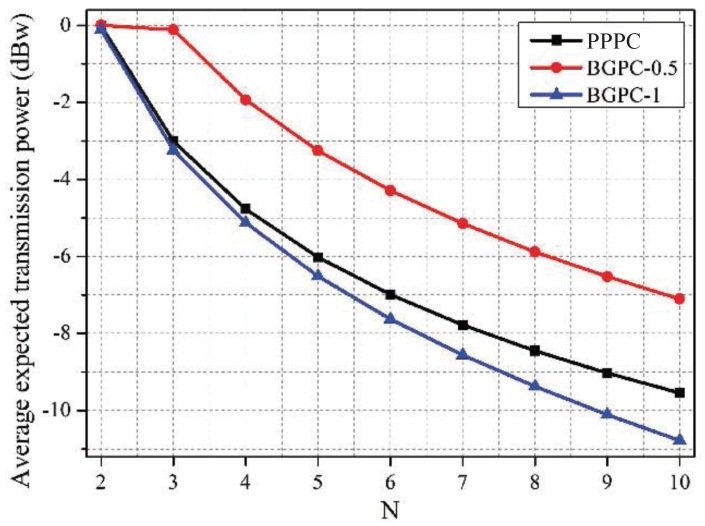
Average expected transmission power vs. *N* under schemes with different pricing mechanisms.

**Figure 11:**
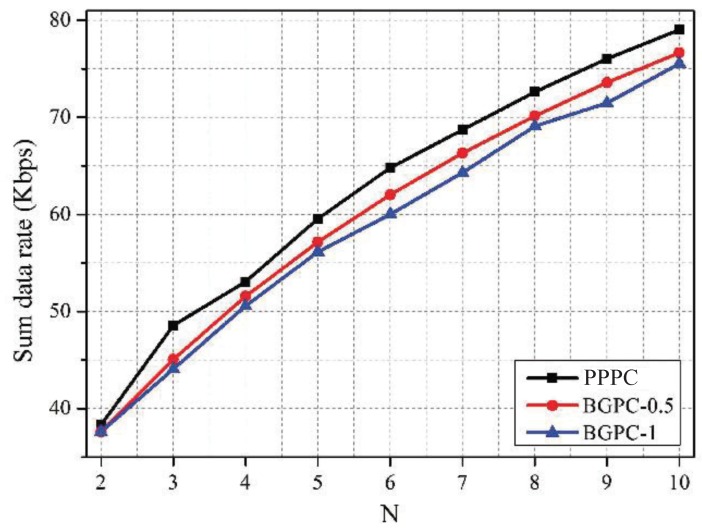
Sum data rate vs. *N* under schemes with different pricing mechanisms.

Figure 9, Figure 10 and Figure 11 depict the comparison between the PPPC scheme and BGPC scheme. The prices of the BGPC scheme were set to 0.5 and 1 in the simulation; these cases are denoted as BGPC-0.5 and BGPC-1.

It can be drawn out that the average price of the PPPC scheme decreased as the number of co-located WBANs increased, as analyzed in Figure 2. Compared with the BGPC-1 scheme, the PPPC scheme achieved a 5.41% higher sum data rate by sacrificing 0.02 W of power. Further, compared with BGPC-0.5, the PPPC scheme obtained a 3.47% higher sum data rate with 62.57% lower transmission power. It can be concluded that the BGPC scheme limited the space for improving the network performance by setting a fixed interference price in advance, while the PPPC scheme with adjustable prices was more flexible and was more energy efficient.

## 6. Conclusions

In this paper, a priority-aware price-based power control scheme was proposed to mitigate the inter-WBAN interference, which was based on the Stackelberg and Bayesian game models. Since the TDMA-based MAC protocol was adopted within each WBAN, while the specific time allocation was private, we assumed there was a virtual player in each WBAN that took the active sensor of the WBAN as its type. Thus, in the game, the coordinators were leaders and set the interference prices, whereas the virtual players were followers and adjusted their transmission powers based on the received prices. There was a non-cooperative game structure at both the leader-level and the follower-level, in which the players aimed to maximize their own expected utilities selfishly. Due to the special features of WBANs, the sensors’ priority factors were considered in the design of the utility functions, and the active probability of each sensor was set to be proportional to its priority factor. Finally, a power control algorithm was designed to obtain the optimal solutions. Extensive simulation results showed that the sensors based on the proposed PPPC scheme could adjust their transmission powers according to their priority levels to improve the timeliness and reliability of critical data transmission and prolong the network lifetime. Moreover, the proposed scheme converged quickly in different scenarios. Furthermore, compared with the OPTIMAL and EVEN schemes, the PPPC scheme achieved a good tradeoff between the network sum data rate and the fairness among sensors. In addition, it was more energy efficient than the existing BGPC scheme. Thus, the proposed PPPC scheme is applicable to mobile WBANs that monitor various physiological parameters with limited energy.

## Figures and Tables

**Table 1:** Notation table.

Symbol	Definition
*N*	Number of co-located WBANs
*m*	Number of sensors in each WBAN
$B_{i}$	$i^{\mathrm{th}}$ WBAN
$V_{i}$	$i^{\mathrm{th}}$ virtual player
$S_{ij}$	$j^{\mathrm{th}}$ sensor in $B_{i}$
$C_{i}$	Coordinator in $B_{i}$
$h_{ij}^{k}$	Channel gain from $S_{ij}$ to $C_{k}$
$p_{ij}$	Transmission power of $S_{ij}$
$P_{\max}$	Maximum transmission power of sensors
$\gamma_{ij}$	SINR of $S_{ij}$
$\varphi_{ij}$	Maximum data rate of $S_{ij}$
*W*	Available bandwidth
$\rho_{i}$	Interference price set by $C_{i}$
$I_{i}$	Interference power suffered by $C_{i}$
$U_{i}^{L}$	Utility of $C_{i}$
$U_{i}^{F}$	Expected utility of $V_{i}$
$u_{ij}$	Utility of $V_{i}$ when $S_{ij}$ is active
$\vartheta_{ij}$	Priority factor of $S_{ij}$
$Pr_{ij}$	Active probability of $S_{ij}$
V	Set of virtual players
$T_{i}$	Type set of $V_{i}$
$P_{i}$	Strategy set of $V_{i}$
$\lambda_{ij}$,$\tau_{ij}$,$\eta_{i}$	Lagrange multiplier

**Table 2:** Simulation parameters.

Parameter	Value
Size of the simulation area	1.6 m × 1.4 m
Size of each WBAN	0.5 m × 0.3 m
The number of sensor nodes in a WBAN	2
The maximum transmission power	0 dBw
The available bandwidth	4 kHz

**Table 3:** Logarithms of the priority factors of the sensors.

$S_{11}$	$S_{12}$	$S_{21}$	$S_{22}$	$S_{31}$	$S_{32}$	$S_{41}$	$S_{42}$	$S_{51}$	$S_{52}$
0.1	0.6	0.5	0.8	0.2	0.7	0.1	0.9	0.5	0
$S_{61}$	$S_{62}$	$S_{71}$	$S_{72}$	$S_{81}$	$S_{82}$	$S_{91}$	$S_{92}$	$S_{101}$	$S_{102}$
0	0.7	0.3	0.5	0.7	0.1	0	0.9	0.8	0.1
